# Model-Based Optimization of Electret–Nanofiber Hybrid Multilayer Filters with Stable Performance Under ISO 29463 Discharge Conditions

**DOI:** 10.3390/polym18141725

**Published:** 2026-07-14

**Authors:** Seunguk Lee, Jeonghyeon Lee, Chanhyun Lee, Sehun Kim, Jinwon Jo, Young Chull Ahn

**Affiliations:** 1 Newrizon Inc., Busan 48228, Republic of Korea; seunguk.lee@newrizon.kr (S.L.); jeonghyeon.lee@newrizon.kr (J.L.); chanhyun.lee@newrizon.kr (C.L.); sehun.kim@newrizon.kr (S.K.); jinwon.jo@newrizon.kr (J.J.); 2 Department of Architectural Engineering, College of Engineering, Pusan National University, Geumjeong-Gu, Busan 46241, Republic of Korea

**Keywords:** nanofiber filter media, electret–nanofiber hybrid filters, model-based optimization, ISO 29463 discharge conditions, slip flow effect, ultrasonic bonding, energy-efficient air filtration

## Abstract

The continuous miniaturization of semiconductor and lithium-ion battery manufacturing processes has intensified the demand for stringent particulate control in cleanrooms while simultaneously increasing the importance of energy efficiency. However, conventional melt-blown (MB) electret filters suffer from severe filtration efficiency degradation under ISO 29463 discharge conditions, whereas glass fiber filters exhibit an excessive pressure drop. To address this trade-off, in this study we propose an electret–nanofiber hybrid multilayer filter and establish a dual-efficiency model for its systematic optimization. The proposed model integrates the slip flow effect of nanofibers and incorporates a structural resistance factor (β = 0.125) derived from the ultrasonic bonding process. Experimental validation demonstrates that the model achieves high predictive accuracy (R^2^ = 0.9995), and the optimized hybrid filter maintains > 98.1% filtration efficiency after ISO 29463-5:2022 discharge testing, markedly outperforming conventional MB filters (41.4%). Furthermore, the hybrid filter exhibits a quality factor (QF) of 0.117, more than twice that of commercial glass fiber filters. These findings demonstrate that the proposed model-based framework provides robust design guidelines for next-generation energy-efficient air filtration systems capable of meeting stringent international standards.

## 1. Introduction

### 1.1. Research Background: Structural Trade-Off Between Cleanroom Air Cleanliness and Energy Efficiency

The manufacturing processes of semiconductors and lithium-ion secondary batteries increasingly require particle control at the nanometer scale as device miniaturization and process integration advance. Consequently, managing airborne particle concentrations in cleanrooms has become a critical factor determining product yield and process reliability. However, maintaining such ultra-clean environments inevitably requires the continuous operation of large-scale heating, ventilation, and air-conditioning (HVAC) systems, which directly leads to substantial energy consumption.

**Figure 1:**
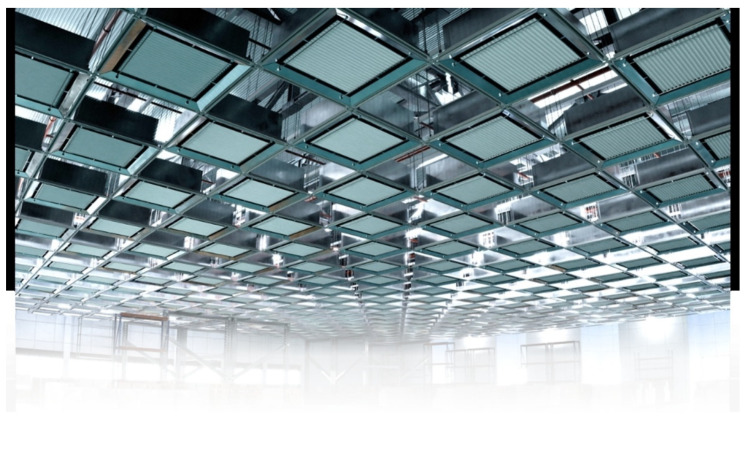
Schematic illustration of FFU (fan filter unit) installation in a semiconductor cleanroom.

In semiconductor cleanrooms in particular, multiple air-handling units (AHUs), including outdoor air conditioners (OACs), are employed for primary filtration, while secondary filtration is achieved through installing and operating thousands to tens of thousands of fan filter units (FFUs). As illustrated in Figure 1, FFUs are typically installed at the ceiling of semiconductor cleanrooms to provide high-efficiency air filtration and maintain the required level of air cleanliness. The pressure drop of high-performance air filters installed in these AHUs and FFUs is directly linked to fan power consumption [1]. Previous measurement-based studies conducted in actual cleanrooms have reported that AHU and FFU system energy consumption strongly depends on the resistance characteristics of the installed filters, and that reducing filter pressure drop is one of the most effective approaches for improving the overall energy efficiency of cleanroom facilities [2]. These findings indicate that pressure drop should be considered a key design parameter in developing air filtration systems.

Cleanroom air filtration differs from general ventilation filtration because even low concentrations of submicron and nanoscale particles can affect yield, process stability, and contamination control in semiconductor and lithium-ion battery manufacturing [3]. Therefore, filter media for cleanroom applications must be evaluated not only by their initial filtration efficiency but also by their pressure drop, post-discharge stability, and ability to control fine particles under long-term operation. Conventional glass fiber filters provide stable mechanical filtration performance but generally impose relatively high pressure drop, whereas electret melt-blown filters exhibit low initial resistance but can suffer from efficiency degradation after charge neutralization. Nanofiber-based filters offer a potential route to improve mechanical capture efficiency while maintaining relatively low pressure drop through nanoscale fiber networks and slip flow effects.

### 1.2. Limitations of Conventional Filter Technologies and ISO 29463 Requirements

Melt-blown (MB) electret filters, widely used in HVAC applications, capture fine particles through electrostatic charges stored on the fiber surfaces, thereby achieving high initial filtration efficiency under relatively low pressure drop conditions. However, they suffer from inherent limitations under real operating environments, where the electrostatic charges on the fiber surfaces gradually dissipate or are shielded due to high humidity, neutral particle loading, or exposure to oil mist, resulting in a significant filtration efficiency deterioration [4]. Previous studies investigating electret filter degradation mechanisms have clearly demonstrated that this efficiency loss originates not from changes in mechanical particle capture capability, but from alterations in the electrical properties of the filter media [5,6].

Reflecting these issues, the reinforced international standard ISO 29463 classifies HEPA and ULPA filters based on the minimum filtration efficiency measured under discharge conditions, in which electrostatic charges are removed using isopropyl alcohol (IPA) vapor during performance evaluation [7]. In particular, ISO 29463-5:2022 [8] defines post-discharge performance testing as an independent evaluation item, thereby establishing filtration performance stability under actual operating conditions as a core criterion for filter classification. As a result, many commercially available MB electret filters fail to satisfy the required performance grades under discharge conditions despite exhibiting high initial efficiencies. In contrast, glass fiber filters rely on purely mechanical particle capture mechanisms that are independent of electrostatic effects, resulting in superior performance stability; however, their inherently high packing density leads to excessive pressure drop, which is disadvantageous from an energy efficiency perspective. Consequently, conventional filtration technologies have not fundamentally resolved the structural trade-off between performance stability and energy efficiency.

### 1.3. Previous Studies: Achievements and Limitations of Model-Based Filter Optimization Approaches

To overcome these limitations, model-based approaches utilizing physical principles rather than empirical design methods have been proposed to optimize filtration performance. Park et al. established filtration efficiency and pressure drop models based on single-fiber theory and experimentally validated them, thereby presenting a systematic optimization framework for metal fiber filters [9]. This study is regarded as a representative example that significantly enhanced the reproducibility and predictability of filter design by interpreting filtration performance as a function of design parameters and underlying physical mechanisms rather than as an isolated experimental outcome.

Meanwhile, in the air filtration field, filter media incorporating fibers with nanometer-scale diameters have attracted considerable attention as promising alternatives to conventional micrometer-scale fiber filters, as they can simultaneously achieve high porosity and large specific surface area, resulting in superior efficiency–pressure drop characteristics. Nanofiber filter media fabricated via electrospinning enable precise filtration performance control through adjustments in filter structure, fiber diameter, and stacking configuration, as systematically summarized in recent review studies [10,11,12,13]. In particular, ultrafine PVDF-based nanofibers have been comprehensively reviewed for their dual contribution as both mechanical capture media and self-polarized electret filter materials, highlighting the inherent versatility of polymeric nanofiber systems for high-performance air filtration [11].

In particular, for filter media composed of nanometer-scale fibers, the conventional assumption of continuum flow is no longer valid, and slip flow effects at the fiber surface play a critical role in determining pressure drop characteristics. Experimental studies on nanofiber filter media have verified that slip flow effects contribute to reducing pressure drop under low-pressure conditions [14,15], and further theoretical and experimental investigations have reported that slip flow corrections become essential as fiber diameter decreases [16,17].

However, most of these studies focused on single-layer or single-material filters and did not directly address electrostatic stability issues or industrial standards such as the discharge conditions specified in ISO 29463. Although some studies experimentally demonstrated that multilayer electret–nanofiber configurations can simultaneously improve aerosol loading performance and filtration efficiency, these studies likewise did not explicitly investigate performance stability under discharge conditions or standard-based evaluations [18,19].

In addition, structural resistance factors arising from filter manufacturing processes, such as ultrasonic bonding, have not been incorporated into existing optimization models; therefore, to apply model-based optimization frameworks to practical cleanroom HVAC systems, extensions that account for more complex filter structures and realistic operating and manufacturing conditions are required.

### 1.4. Study Objectives and Approach

The theoretical starting point of this study is the single-fiber-based filtration efficiency–pressure drop model and the associated filter optimization framework proposed by Park et al. [9]. This framework transformed conventional filter design approaches, which relied heavily on empirical trial-and-error processes, into a model-based optimization methodology by formulating filter performance as a function of design parameters and physical mechanisms and validating the model experimentally.

However, the framework proposed by Park et al. [9] was developed for single-material filters and did not account for several critical factors relevant to real industrial environments, including electrostatic charge decay in electret filters, the discharge conditions required by ISO 29463, slip flow effects occurring at nanometer-scale fibers, and structural resistance introduced by manufacturing processes such as ultrasonic bonding.

In this study, the model-based optimization concept proposed by Park et al. [9] is extended to a hybrid multilayer filter structure. The electret fiber and nanofiber layers are separately modeled to independently describe their respective filtration mechanisms and pressure drop characteristics, and the two models are integrated into a dual-efficiency model. For the nanofiber layer, the reduction in aerodynamic drag due to slip flow effects is incorporated to improve the pressure drop prediction accuracy. Furthermore, structural resistance arising from ultrasonic bonding is parameterized and included in the overall pressure drop formulation, enabling filter design that reflects actual manufacturing conditions.

Although previous studies have reported PVDF-based nanofiber filters, electret filtration media, and multilayer filter structures, most have focused primarily on initial filtration performance, pressure drop reduction, or material fabrication. In contrast, the present study focuses on the post-discharge performance required under ISO 29463-5 conditions and develops a model-based optimization framework for electret–nanofiber hybrid multilayer filters. The main contribution of this work is the integration of mechanically retained filtration efficiency after discharge, slip flow-corrected pressure drop modeling, and ultrasonic-bonding-induced structural resistance into a practical hybrid filter design.

Our objective in this study is to derive a hybrid filter structure that simultaneously satisfies high filtration efficiency and low pressure drop under ISO 29463 discharge conditions using the proposed extended optimization framework, and to experimentally verify its validity. By doing so, we aim not only to predict filtration performance but also to provide practical design guidelines that enable the simultaneous achievement of energy efficiency and performance stability.

## 2. Theoretical Background

In this section, we systematically review the fundamental theories governing filtration performance in order to establish the physical validity of the electret–nanofiber hybrid multilayer filter and the dual-efficiency model proposed in this study. Specifically, the discussion sequentially addresses single-fiber filtration theory, slip flow effects occurring at nanometer-scale fibers, and electret charge decay mechanisms. Through this theoretical framework, in the present study we aim to clearly demonstrate that the proposed optimization approach is based on validated physical models rather than empirical design methodologies.

### 2.1. Single-Fiber Filtration Theory and the Position of the Park et al. [9] Model

**Figure 2:**
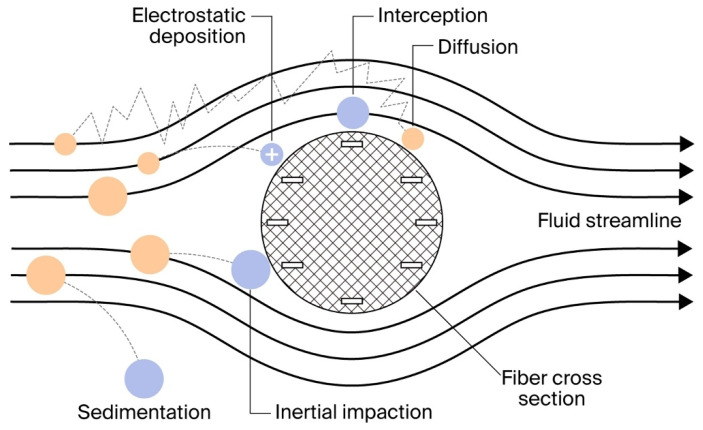
Schematic of particle capture mechanisms based on single-fiber filtration theory (adapted from [21]).

The fundamental principle of fibrous filtration is explained by superimposing the particle capture mechanisms occurring at the individual fiber level. According to classical single-fiber filtration theory, as illustrated in Figure 2, the overall mechanical collection efficiency of a filter is determined by diffusion, interception, inertial impaction, and gravitational settling, with each mechanism assumed to act independently. This theoretical framework was systematically established through the textbook studies of Brown and Hinds [20,21].

Park et al. [9] applied this single-fiber theory to metal fiber filters and proposed a formulation for the overall mechanical collection efficiency by combining the individual mechanical capture mechanisms in a multiplicative form, which was subsequently validated through experimental measurements. Accordingly, the mechanical collection efficiency of a single fiber, denoted as $E_{mech}$, is defined as follows:

(1) $$E_{mech}=1-(1-E_{D})(1-E_{I})(1-E_{R})(1-E_{G})$$

Here, $E_{D}$, $E_{I}$, $E_{R}$, and $E_{G}$ represent the collection efficiencies due to diffusion, inertial impaction, interception, and gravitational settling, respectively. This formulation is based on the assumption that each capture mechanism independently acts probabilistically. Park et al. [9] demonstrated through experimental results that this assumption is sufficiently valid within the design range of metal fiber filters. In the present study, this model is adopted as a theoretical starting point and extended to a hybrid multilayer filter structure combining electret fibers and nanofibers.

In particular, the collection efficiency due to diffusion, $E_{D}$, is expressed as a function of the Péclet number (Péclet number, Pe) and is generally approximated using the following form [20,21]:

(2) $$E_{D}\propto Ku^{-1/3}\cdot Pe^{-2/3}$$

This indicates that diffusion-based particle capture increases rapidly as the fiber diameter decreases, thereby providing a theoretical basis for nanometer-scale fiber advantages in fine particle filtration.

### 2.2. Slip Flow Effects in Nanofiber Filter Media

Conventional filtration theories assume a no-slip boundary condition in which the fluid velocity at the fiber surface is zero. However, when the fiber diameter ($d_{f}$) decreases to the nanometer scale and becomes comparable to the mean free path of air molecules (λ≈65 nm), this assumption is no longer valid. As the Knudsen number ($Kn=2\lambda /d_{f}$) increases, gas molecule slip flow occurs at the fiber surface, which reduces the aerodynamic drag around the fiber and leads to a significant decrease in pressure drop [14].

The relevance of slip flow can be evaluated using the Knudsen number, defined as Kn = 2λ/df, where λ is the mean free path of air molecules and df is the fiber diameter. Under standard atmospheric conditions, λ is approximately 65 nm. Therefore, when the fiber diameter decreases to the sub-100 nm range, the characteristic fiber dimension becomes comparable to the molecular mean free path, and the no-slip boundary condition becomes increasingly invalid. In this regime, slip flow correction is required to avoid overestimating aerodynamic drag and pressure drop.

As shown in Table 1, the Knudsen number increases rapidly as the fiber diameter decreases, indicating that slip flow effects become increasingly significant in nanofiber media, particularly when the fiber diameter approaches or falls below 100 nm.

Bao et al. [14] experimentally verified the existence of slip flow effects by precisely measuring nanofiber filter pressure drop under low-pressure conditions. Subsequently, Choi et al. [15] and Kwak et al. [17] quantified the slip flow effect as a function of fiber diameter and demonstrated that slip flow correction is essential in designing nanofiber filters.

Based on these previous studies, in the present work we employ a modified pressure drop model in which the Cunningham correction factor ($C_{c}$) is incorporated into the Davies model to account for slip flow effects in pressure drop prediction.

(3) $$\Delta P_{nano}=\frac{\Delta P_{Davies}}{1+Kn\left(1.257+0.4exp(\frac{-1.1}{Kn})\right)}$$

Through this approach, the limitations of conventional continuum-flow-based models can be addressed, as they tend to overestimate the actual pressure drop occurring in nanofiber layers.

Because electrospun nanofiber layers generally exhibit a distribution of fiber diameters rather than a single uniform value, the model prediction should be interpreted based on the representative or measured fiber diameter distribution. The contribution of slip flow may be reduced when a substantial fraction of larger-diameter fibers is present.

### 2.3. Electret Charge Decay and the Dual-Efficiency Concept Based on ISO 29463-5

Electret filters capture fine particles effectively through Coulombic and dielectrophoretic forces generated by electrostatic charges stored on fiber surfaces. However, such electrostatic capture mechanisms are gradually attenuated under practical operating conditions, including neutral particle loading, high-humidity environments, exposure to oil mist, and contact with organic solvents. Cai et al. [5] theoretically and experimentally demonstrated that charge dissipation in electret filters during neutral particle loading leads to a corresponding reduction in filtration efficiency, which originates from changes in the electrical properties of the filter media. Beyond particle-loading-induced decay, Lee and Kim [22] further showed that the intrinsic dielectric constant and surface energy of the polymer matrix govern electret filter charge retention capacity, with high-dielectric-constant materials such as PVDF being particularly susceptible to charge loss under thermal and humidity aging. Lavoie et al. [23] additionally demonstrated that even with charge-protection additives, exposure to alcohol or oily aerosols inevitably degrades electrostatic capture performance in polypropylene-based electret media, underscoring the structural inevitability of this limitation.

To institutionally reflect these real operating conditions, the recently revised international standard ISO 29463-5:2022 specifies filtration performance evaluation under discharge conditions as an independent test procedure in which filter media electrostatic charges are removed using isopropyl alcohol (IPA) vapor [8]. This revision signifies a shift from conventional evaluation approaches focused on initial filtration efficiency toward performance stability under actual operating conditions as a core criterion for filter classification.

In the present model, the post-discharge condition was represented by setting the electrostatic collection efficiency to $E_{elec}$ = 0. This assumption does not imply the complete physical absence of all residual charges in the filter media. Rather, it is a conservative modeling approach consistent with the purpose of ISO 29463-5 discharge testing, in which IPA vapor treatment is used to neutralize the electret contribution and evaluate the minimum filtration performance after charge removal. Under this condition, the remaining filtration efficiency is attributed primarily to mechanical capture mechanisms such as diffusion, interception, inertial impaction, and gravitational settling. Therefore, the assumption $E_{elec}$ = 0 provides a conservative lower-bound design basis for evaluating post-discharge filtration performance.

(4) $$E_{total}=1-(1-E_{mech})(1-E_{elec}),{\;E}_{elec}\to 0\;(\mathrm{discharged}\;\mathrm{state})$$

Although PVDF can exhibit electroactive or electret-like behavior depending on processing and poling conditions, the PES/PVDF nanofiber layer in this study was not treated as an actively poled electret layer. Any residual electrostatic contribution from the PVDF-containing nanofiber layer after discharge was therefore not credited in the model, making the post-discharge prediction conservative.

In this study, building upon the single-fiber-based optimization concept proposed by Park et al. [9], a hybrid multilayer filter design framework is developed by integratively considering a nanofiber layer incorporating slip flow effects and a mechanically robust capture structure that maintains performance under discharge conditions. Through this approach, a theoretical foundation is established to design hybrid multilayer filters that comply with the latest international standards.

## 3. Materials and Methods

In this study, a proprietary numerical simulation algorithm was employed to predict the filtration efficiency and pressure drop of multilayer filter media. The model consists of a three-step procedure: (i) calculating mechanical efficiency based on single-fiber filtration theory, (ii) applying electrostatic charge decay conditions, (iii) integrating multilayer structures with correction for ultrasonic bonding effects.

The model input parameters include fiber diameter, packing density, filter thickness, face velocity, stacking configuration, and target filtration grade. The output parameters are the initial and post-discharge filtration efficiencies, pressure drop, and quality factor (QF). Numerical optimization is performed with the following objective function: maximizing the QF while satisfying the minimum filtration efficiency required under the ISO 29463-5:2022 discharge condition.

### 3.1. Numerical Model Formulation

#### 3.1.1. Dual-Efficiency Model

To quantitatively decouple the contribution of electrostatic capture, a dual-efficiency model was introduced in which the total filtration efficiency was defined as a combined function of mechanical efficiency and electrostatic efficiency.

(5) $$E_{total}=1-(1-E_{mech})(1-E_{elec})$$

Under the discharge test conditions specified in ISO 29463-5:2022, the electrostatic capture effect can be assumed to be nearly eliminated. Accordingly, in this study, the electrostatic collection efficiency under post-discharge conditions was set to $E_{elec}=0$, enabling us to predict the lower bound of the mechanical collection efficiency required in filter design and allowing for a quantitative evaluation of the nanofiber layer’s role.

#### 3.1.2. Definition of the Ultrasonic Bonding Factor

The total pressure drop of a multilayer filter can be calculated as the sum of the individual filter layer pressure drops. However, the ultrasonic bonding process induces localized fiber melting and a reduction in the effective filtration area, so to account for these effects, an ultrasonic bonding factor (β) was introduced in this study.

(6) $$\Delta P_{total}=\left(\sum_{i=1}^{n}\Delta P_{i}\right)(1+\beta )$$

The bonding factor, β, was experimentally derived from the difference in the pressure drop between the simply stacked structure and ultrasonically bonded final product; the validity of this factor was verified in Section 4.1.

### 3.2. Experimental Materials and Filter Fabrication

Nanofiber filter media were fabricated using an electrospinning process, with the average fiber diameter controlled within the range of 50–200 nm. The nanofibers were produced based on PES/PVDF polymers, while polypropylene (PP) melt-blown media were used as the electret fiber layer. The overall multilayer structure was composed in the following order: a PET support layer, an electret fiber layer, and a nanofiber layer. The stacked multilayer structure was bonded without using adhesives by employing a roll-to-roll ultrasonic bonding process. Commercially available H13-grade glass fiber filters and melt-blown filters were used as reference samples for comparison.

**Figure 3:**
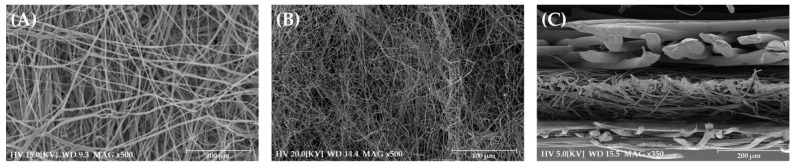
SEM images of (**A**) electret (surface view), (**B**) nanofiber (surface view), (**C**) electret–nanofiber hybrid filter (cross-section view).

The nanofiber layer was fabricated by electrospinning a PES/PVDF polymer solution onto a PET support substrate. The key electrospinning parameters, including polymer composition, solvent system, applied voltage, feed rate, tip-to-collector distance, and basis weight, are summarized in Table 2. As shown in Figure 3, the surface morphologies of the electret and nanofiber layers and the cross-sectional structure of the electret–nanofiber hybrid filter were examined by SEM.

### 3.3. Performance Characterization

Filtration efficiency and pressure drop were measured using a TSI 8130 automated filter tester. A sodium chloride (NaCl) aerosol with a mass median diameter of 0.26 μm was used as the test particle, and the face velocity was set to 5.3 cm/s. The commercial glass fiber and e-PTFE reference media used for quality factor comparison were evaluated under the same face velocity and aerosol test conditions as the hybrid filter. Their filtration efficiency, pressure drop, and quality factor were compared using identical measurement protocols to ensure consistency. Compliance with ISO 29463-5 was evaluated by exposing the filters to a saturated IPA vapor environment for 24 h, followed by conducting performance measurements after sufficient stabilization time had passed. The microstructure of the filter media was examined using scanning electron microscopy (SEM).

## 4. Results and Discussion

### 4.1. Model Validation

#### 4.1.1. Ultrasonic Bonding Factor and Accuracy of Pressure Drop Prediction

To validate the accuracy of pressure drop prediction, experiments were conducted using three structural configurations: a PET support plus electret fiber layer (MB), a nanofiber layer (NF), and an ultrasonically bonded hybrid layer (MB+NF). The additional structural resistance caused by ultrasonic bonding was then quantified by comparing the pressure drop of the simply stacked structure with that of the ultrasonically bonded hybrid structure. Five repeated measurements were performed for each configuration, and the mean ± standard deviation of the pressure drop measurements and the calculated ultrasonic bonding factor are summarized in Table 3. The pressure drop of the ultrasonically bonded hybrid structure exceeded the simple additive pressure drop of the individual layers, indicating the presence of manufacturing-induced structural resistance. Based on the measured pressure drop increase, the ultrasonic bonding factor was determined as approximately β = 0.125.

(7) $$\beta =\frac{\left({∆P}_{bonded}-{∆P}_{simple\;stack}\right)}{{∆P}_{simple\;stack}}$$

Although the bonding factor calculated from the measured mean values was 0.122, the value was expressed as approximately (β = 0.125) in the model to represent a rounded structural resistance factor based on the measured pressure drop increase.

**Figure 4:**
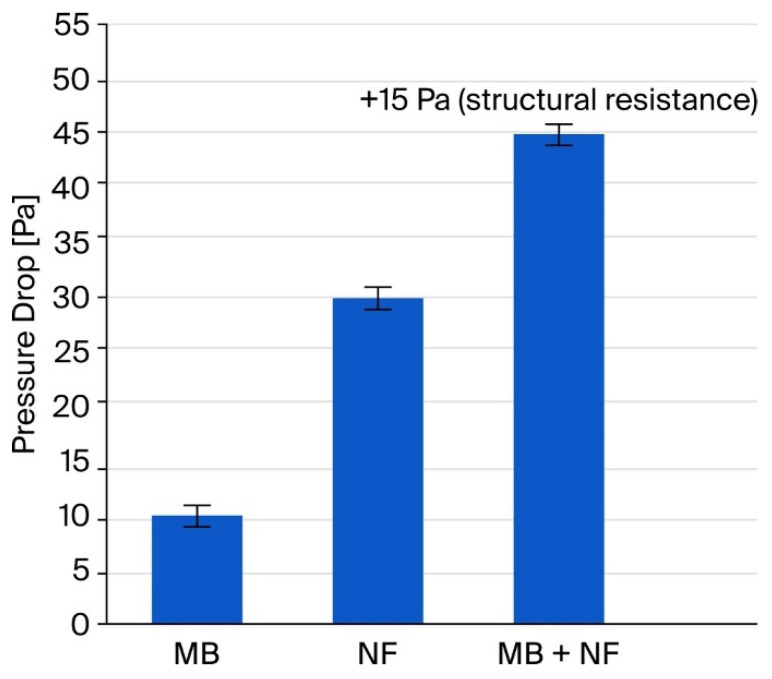
Effect of ultrasonic bonding on the pressure drop of the hybrid filter structure. The bonded hybrid structure exhibited an additional pressure drop, indicating bonding-induced structural resistance corresponding to approximately β = 0.125 in the model.

Figure 4 illustrates that the ultrasonically bonded structure pressure drop deviates from the simple additive trend of the individual layers. The observed pressure drop increase in the bonded structure cannot be explained solely by the additive resistance of individual layers, indicating the presence of additional structural effects induced during bonding. Recent investigations on ultrasonically bonded polypropylene melt-blown nonwovens have shown that the bonding process locally densifies the fibrous network through adjacent inter-fiber contacts, which alters the effective porosity and increases aerodynamic resistance beyond the simple sum of individual layer contributions [24]. This finding supports the introduction of a structural resistance factor in the present model.

**Figure 5:**
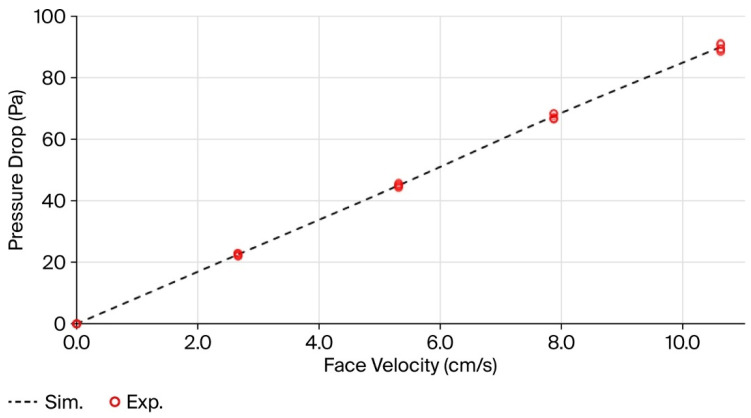
Comparison between simulated and experimental pressure drop values at different face velocities.

Using the corrected model, the simulated pressure drop values were compared with the experimentally measured value of the fabricated filter media. As shown in Figure 5, a very strong correlation was observed between the simulated and experimental values across the entire range of tested face velocities. Based on five repeated samples at each velocity, the coefficient of determination was evaluated to be $R^{2}=0.9995$.

This excellent agreement demonstrates that the proposed model accurately captures both the slip flow-induced drag reduction in the nanofiber layer and the additional resistance introduced by ultrasonic bonding. Furthermore, the strong correlation confirms the robustness and predictive reliability of the model, even when applied to a fully bonded multilayer filter structure rather than an idealized stacked configuration.

In addition to the coefficient of determination, prediction error metrics were calculated to evaluate the accuracy of the pressure drop model. Based on the nonzero velocity data, the model exhibited low prediction errors, with MAE = 0.685 Pa, RMSE = 0.779 Pa, and MAPE = 1.26%. These results confirm that the proposed model accurately predicts the pressure drop behavior of the bonded hybrid filter over the tested face velocity range.

#### 4.1.2. Validation of Filtration Efficiency Under Discharge Conditions

The filtration efficiencies before and after IPA vapor treatment were compared. While the commercial melt-blown electret filter exhibited a substantial reduction in efficiency after discharge, the hybrid filter proposed in this study maintained an efficiency exceeding 98.1% under the same conditions, indicating that the nanofiber layer provides stable mechanical particle capture performance even under discharge conditions.

As summarized in Table 4, the post-discharge filtration performance of the hybrid filter is governed primarily by mechanically driven capture mechanisms. Under discharge conditions, the electrostatic contribution becomes negligible, causing the total filtration efficiency to converge toward the mechanical efficiency limit defined by the filter structure. The high efficiency retained after discharge therefore confirms that the nanofiber layer effectively compensates for the electrostatic capture loss, enabling stable performance in compliance with ISO 29463-5 requirements.

### 4.2. Applicability of the Model Under Various Industrial Conditions

To evaluate the applicability of the proposed model under various practical industrial conditions, including automotive, power plant, and air purifier applications, the simulated and experimentally measured values were compared. Good agreement was observed across all tested conditions, confirming that the model remained applicable under a wide range of operating conditions. The comparison results are summarized in Table 5.

Overall, the pressure drop behavior predicted by the model consistently followed experimental trends despite variations in operating conditions and application requirements. This robustness can be attributed to the physics-based model formulation, which incorporates slip flow effects and structural resistance independently of specific application environments. As a result, the model is not limited to a single filtration scenario but extends to diverse industrial applications with different airflow and resistance requirements.

### 4.3. Performance and Energy Efficiency Analysis Compared with Conventional Filter Media

The quality factor (QF) is defined by the following equation:

(8) $$QF=-\frac{ln(1-\eta )}{\Delta P}$$

The optimized hybrid filter exhibited a significantly lower pressure drop and a higher QF value compared with conventional glass fiber and e-PTFE filters.

**Figure 6:**
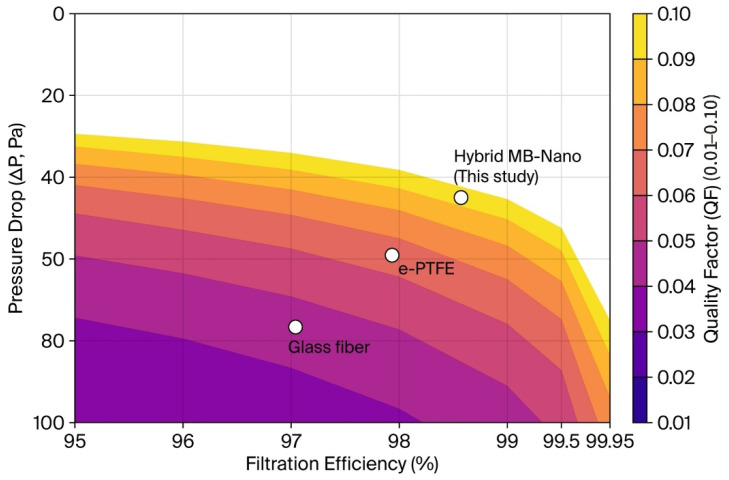
Comparison of quality factors (QFs) for the proposed hybrid filter and conventional reference filter media.

Figure 6 highlights that the superior QF of the hybrid filter is achieved not merely through enhanced filtration efficiency but primarily through a substantial pressure drop reduction. While glass fiber and e-PTFE filters rely on dense fiber packing to ensure a stable mechanical capture, this approach inevitably results in increased aerodynamic resistance. In contrast, the hybrid filter leverages slip flow-induced drag reduction in the nanofiber layer to maintain high mechanical capture efficiency without incurring a pressure drop penalty. This balance between efficiency and resistance underpins the improved energy performance of the hybrid filter.

**Figure 7:**
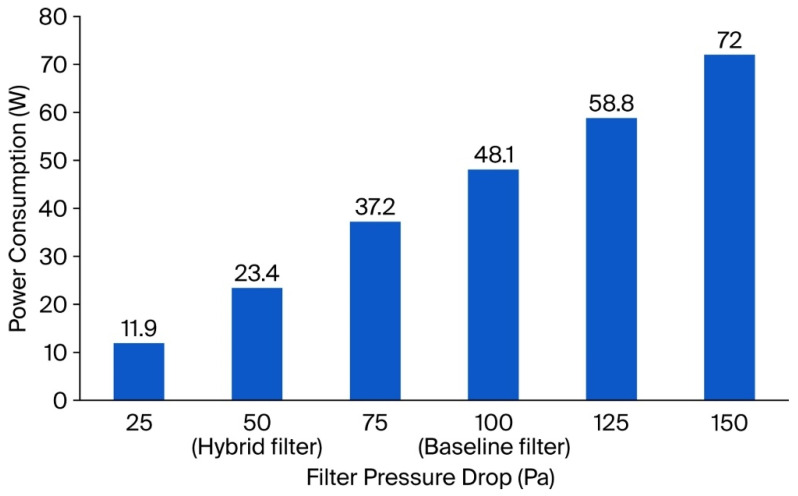
Experimental relationship between filter pressure drop and FFU power consumption under simplified pressure-drop-based scaling.

As shown in Figure 7, a reduction in filter pressure drop is directly associated with a decrease in fan power consumption [1]. Based on the proportional relationship $P_{elec}\propto \;Q\Delta P$, applying the proposed hybrid filter to fan filter units (FFUs) is expected to provide potential power-saving benefits under simplified pressure-drop-based scaling. In this study, the estimated FFU power saving potential was approximately 52%; however, this value should be interpreted as an indicative estimate rather than a universal system-level prediction. Actual energy savings may vary depending on the FFU fan curve, airflow control strategy, operating airflow rate, filter loading condition, installation configuration, and facility-level control logic. Therefore, the present analysis demonstrates the potential energy-saving implication of pressure drop reduction, while full-scale FFU validation is required for quantitative system-level confirmation.

## 5. Conclusions

In this study, an electret–nanofiber hybrid multilayer filter designed to comply with ISO 29463-5 discharge requirements was developed, and a numerical model was proposed to optimize its filtration performance. By incorporating slip flow effects and an ultrasonic bonding factor (β = 0.125), the model demonstrated an exceptionally high predictive accuracy, as confirmed by the strong agreement between the simulated and experimental results (R^2^ = 0.9995).

The optimized hybrid filter maintained a high filtration efficiency of > 98.1% even under discharge conditions, indicating that mechanically driven capture mechanisms provided by the nanofiber layer effectively compensate for the loss of electrostatic effects (which dropped to 41.4% in conventional MB filters).

Compared with conventional glass fiber and e-PTFE filters, the proposed hybrid filter achieved a superior quality factor (QF = 0.117), primarily through a substantial reduction in pressure drop. Under simplified fan power scaling, this pressure drop reduction indicates the potential for FFU power-saving benefits; however, the estimated value of approximately 52% should be interpreted as an indicative estimate rather than a universal system-level prediction.

Although the hybrid filter demonstrated stable post-discharge filtration efficiency under ISO 29463-5 conditions, long-term durability under continuous particle loading, cyclic humidity exposure, oil mist exposure, and repeated discharge-related stress was not fully evaluated in this study. These factors may affect pressure drop evolution, particle loading behavior, and mechanical integrity during extended operation. Therefore, long-term loading tests and environmental durability evaluations will be required in future work to further validate the practical applicability of the hybrid filter in cleanroom environments.

These findings demonstrate that the proposed model-based design framework provides a practical guideline for developing next-generation cleanroom air filters that simultaneously satisfy stringent performance stability requirements and energy efficiency demands.

## Figures and Tables

**Table 1:** Relationship between fiber diameter and Knudsen number.

Fiber Diameter, d_f_ (nm)	Knudsen Number, Kn *	Interpretation
500	0.26	Slip correction relevant but weak
200	0.65	Slip flow relevant
100	1.3	Slip flow dominant
50	2.6	Strong slip flow effect
30	4.33	Strong slip flow effect

* Kn was calculated as Kn = 2λ/d_f, where λ = 65 nm.

**Table 2:** Electrospinning parameters used for nanofiber layer fabrication.

Parameter	Value	Parameter	Value
Polymer composition	PES/PVDF polymers	Feed rate	3.2 μL/min
Solvent system	DMAc/Acetone	Substrate	Thermal-bonded support media
Electrospinning voltage	22–27 kV	Target fiber diameter	50–150 nm
Tip-to-collector distance	10–12 cm	Basis weight	30 GSM
Collector speed/line speed	20–35 mm/s	Temperature/ Relative Humidity	20.5–24.5 °C/ 30–50% RH

**Table 3:** Determination of the ultrasonic bonding factor based on repeated pressure drop measurements.

Configuration	Mean ΔP (Pa)	SD (Pa)	Note
Case A: PET support + electret layer	10.06	0.29	*n* = 5
Case B: nanofiber layer	29.98	0.19	*n* = 5
Simple stack: A + B	40.04	-	Calculated
Case C: Ultrasonically bonded hybrid structure	44.94	0.27	*n* = 5
β from measured means	0.122	-	(44.94 − 40.04)/40.04

**Table 4:** Comparison of filtration efficiencies before and after discharge and corresponding efficiency retention for different filter media.

Filter	Initial η (%)	Post-Discharge η (%)	Retention * (%)
MB electret (E11)	96.7 ± 0.30	14.9 ± 5.6	15.4
MB electret (H13)	99.99 ± 0.02	41.4 ± 3.1	41.4
Hybrid (MB + NF)	99.2 ± 0.20	98.1 ± 0.4	98.9

* Retention (%) was calculated as (Post-discharge η/Initial η) × 100.

**Table 5:** Comparison of simulated and experimental performance under various application conditions.

Application	Requirement (η/ΔP)	Simulation (η/ΔP)	Experiment (η/ΔP)
Automotive filter (A)	≥98.5%/≤80 Pa	99.54%/75 Pa	98.50%/72 Pa
Automotive filter (B)	≥85%/≤30 Pa	88.85%/22 Pa	90.74%/24 Pa
Power plant filter	≥99.95%/≤120 Pa	99.99%/115 Pa	99.97%/118 Pa
Air purifier filter	≥95%/≤50 Pa	97.85%/48 Pa	96.60%/45 Pa

All measurements were performed at a fixed face velocity of 5.3 cm/s using the same aerosol test protocol.

## Data Availability

The data presented in this study are available within the article. Additional experimental data, simulation results, and supporting materials are available from the corresponding author upon reasonable request.

## Abbreviations

The following abbreviations are used in this manuscript:

MB	Melt-Blown
NF	Nanofiber
QF	Quality Factor
HVAC	Heating, Ventilation, and Air-conditioning
AHUs	Air-Handling Units
OAC	Outdoor Air Conditioner
FFU	Fan Filter Unit
HEPA	High-Efficiency Particulate Air
ULPA	Ultra-Low-Penetration Air
IPA	Isopropyl Alcohol
β	Structural Resistance Factor
Pe	Péclet Number
η	Filtration Efficiency
